# Deterministic Edge-Controlled Precision Fertigation System with Spatial Task Scheduling and Hardware–Software Safety Interlock

**DOI:** 10.3390/s26134289

**Published:** 2026-07-06

**Authors:** Ziheng Wang, Jiahui Chen, Hongjian Zhao, Bing Wei

**Affiliations:** 1 Beijing Research Institute of Automation for Machinery Industry Co., Ltd., Beijing 100120, China; wangziheng0922@163.com (Z.W.); zhaohj95@163.com (H.Z.); 2 China Academy of Machinery Wuhan Research Institute of Materials Protection Co., Ltd., Wuhan 430030, China; jiahui_chen0523@foxmail.com

**Keywords:** precision irrigation, edge computing, Kalman filtering, adaptive control, robotic manipulator, variable-rate fertigation, RS485 sensors, hardware–software interlock

## Abstract

Cloud-dependent irrigation platforms can support remote monitoring, but their use in precision fertigation is limited when local decisions must be made quickly and reliably. Network delay, temporary disconnection, and the use of single-point measurements may all reduce the ability of a system to respond to spatial variation in soil moisture and nutrient demand. In this work, an edge-controlled precision fertigation system was developed by combining multi-parameter soil sensing, spatial task scheduling, and a 6-DOF robotic manipulator. The ESP32 controller runs a preemptive FreeRTOS scheduler, allowing sensor acquisition, inverse-kinematics calculation, and pump actuation to be handled as separate tasks. A Kalman filter was used to smooth soil moisture measurements, and a hysteresis-based control strategy was adopted to reduce false triggering and repeated pump switching. To improve fertigation safety, a hardware–software interlock was added so that fertilizer delivery is always accompanied by water delivery. Hardware-in-the-Loop simulation and a 14-day field deployment were used to evaluate the system. The controller achieved an end-to-end latency of less than 38 ms and maintained operation during network interruptions through cached local parameters. After calibration, the robotic end-effector positioning error was reduced to ±2.4 mm. The hysteresis strategy lowered daily pump cycling by 71%. Based on prototype duty-cycle data and seasonal extrapolation, the projected seasonal water use and fertilizer demand were 44% and 38% lower, respectively, than those estimated for a uniform application. These values should be interpreted as model-based projections rather than direct season-long measurements. During 72 h of continuous operation, no Modbus faults were observed, and RTOS heap fragmentation remained stable. Overall, the results suggest that edge-based deterministic control can provide a practical route for precision fertigation where both spatial variability and intermittent connectivity must be considered.

## 1. Introduction

### 1.1. Research Background

Precision irrigation and fertilization are essential strategies for improving resource-use efficiency and promoting sustainable production in facility agriculture. However, conventional open-loop or timer-based control systems generally lack real-time feedback from the root-zone environment, which can result in excessive water consumption, nutrient leaching, and unstable crop growth conditions. Although low-cost soil sensors provide a practical basis for distributed field monitoring, their measurements are often affected by high-frequency noise, temperature- and salinity-induced drift, and response hysteresis [1]. If pump actuation is triggered directly by raw sensor readings, frequent threshold crossing may cause repeated switching, thereby increasing electromechanical wear and reducing actuator service life [2]. In addition, single-point monitoring cannot adequately characterize the spatial heterogeneity of soil moisture and macronutrient distribution, leading to uniform blanket irrigation or fertilization that may further aggravate resource losses and limit crop-yield uniformity [3].

The integration of edge computing, multi-node sensing, and robotic actuation provides a promising route for site-specific variable-rate application (VRA). Recent studies have attempted to address these limitations through advanced robotic and computing architectures. For instance, Cheng et al. [4] demonstrated that mobile manipulators can significantly extend the operational workspace in greenhouse environments compared to fixed-base systems, although coordinating large-scale movement with high-precision end-effector control remains a challenge. Furthermore, to overcome the latency issues of cloud-dependent systems, Yu et al. [5] proposed an edge-cloud collaborative framework for irrigation, proving that migrating control logic to the edge can drastically reduce response times.

However, existing solutions often lack a unified design that combines macro-micro spatial coordination with deterministic edge control and multi-modal agronomic decision-making. Consequently, implementing deterministic control, safety-critical interlocks, and spatial task scheduling on resource-constrained microcontrollers while maintaining agronomic reliability remains a considerable engineering challenge.

### 1.2. Current Challenges

Although agricultural IoT has developed rapidly in recent years, several practical issues still limit its use in precision fertigation. One important problem is that irrigation and fertilization are often controlled as two separate processes. In such systems, fertilizer may be delivered without sufficient synchronous water supply, especially when concentrated nutrient solution is used. This can increase the possibility of local salt accumulation, osmotic stress, or fertilizer-induced root injury under unsuitable dosing conditions [6].

Another difficulty lies in the edge-side implementation of real-time control. A fertigation system that considers agronomic safety must process multi-parameter sensor data, schedule spatial tasks, and calculate robotic motion commands within a limited response time. These functions are demanding for low-power microcontrollers. If they are implemented in a conventional super-loop program, sensor polling delays, RS485 timeouts, or Wi-Fi interruptions may block other tasks and lead to unstable response latency. In more severe cases, faults in one peripheral module may propagate to the whole control loop, reducing the reliability needed for autonomous field operation [7,8].

System validation is also a concern. Many reported irrigation or fertigation prototypes are tested only through offline simulation or short-duration experiments. Before field deployment, however, the edge firmware, soil-response model, actuator logic, and safety interlock should be examined together under repeatable conditions. Hardware-in-the-Loop (HIL) testing provides a practical way to bridge this gap, but its use in precision fertigation studies remains limited. The lack of such pre-deployment verification can increase field-testing uncertainty and make it difficult to compare results across different systems [9,10].

### 1.3. Contributions of This Work

To overcome the above limitations, this study develops an edge-controlled precision fertigation system that combines multi-point soil sensing, robotic spatial delivery, and water–fertilizer safety interlocking. The system was designed to keep the time-critical control loop on the edge device, while using the cloud only for parameter updates, data storage, and visualization. The main contributions are as follows:

1. **Edge-side deterministic control based on FreeRTOS:** A dual-core ESP32 controller was configured with a preemptive FreeRTOS scheduler [11]. Sensor polling, inverse-kinematics calculation, relay actuation, and telemetry uploading were assigned to separate tasks with different priorities. This arrangement reduced blocking between peripheral operations and kept the end-to-end control latency below 38 ms in the tested cases. During network interruptions, the controller continued operation using cached local parameters.
2. **Robotic spatial fertigation with node-level task scheduling:** A 6-DOF robotic manipulator was used to guide the nozzle to target sensing nodes. The joint angles were calculated by an embedded damped least-squares in-verse-kinematics solver. Together with a priority-based task queue and a 15% hysteresis deadband, the system performed site-specific water and fertilizer delivery and reduced daily pump cycling by 71% compared with the fixed-threshold baseline.
3. **Water–fertilizer interlock for safer nutrient delivery:** A hardware–software interlock was introduced to enforce the control rule $P_{\mathrm{fert}}=\mathrm{ON}⟹P_{\mathrm{water}}=\mathrm{ON}$, under this rule, the fertilizer pump cannot be activated independently of the water pump. This design reduces the risk of applying concentrated nutrient solution without dilution and follows the safety-oriented design principles of ISO 18497-1:2024 [12] for agricultural machinery.
4. **Validation from HIL simulation to prototype testing:** A UART–Python digital twin was built to evaluate state transitions, interlock behavior, and task scheduling before physical deployment. The HIL results were then examined using a 14-day prototype test. The system achieved an end-effector positioning accuracy of ±2.4 mm and no Modbus faults during 72 h of continuous operation. In addition, duty-cycle-based seasonal extrapolation suggested potential reductions of 44% in water use and 38% in fertilizer demand compared with uniform application. The firmware and analysis scripts were made publicly available to support reproduction and further testing.

This study focuses on the following research questions: **RQ1:** How can a macro-micro collaborative architecture overcome the workspace limitations of traditional fixed-base manipulators? **RQ2:** How can edge-cloud collaboration ensure deterministic control latency and safety interlocks under network instability? **RQ3:** How can multi-modal perception (vision + soil sensors) improve the early warning accuracy for crop stress compared to single-modal systems?

### 1.4. Paper Organization

The remainder of this paper is organized as follows. Section 2 describes the overall edge-cloud collaborative architecture, hardware implementation, and communication protocol design of the proposed precision fertigation system. Section 3 presents the core control methodology, including Kalman-filter-based state estimation, adaptive threshold generation, spatial task scheduling, embedded inverse kinematics, and the hardware–software safety interlock mechanism. Section 4 describes the Hardware-in-the-Loop (HIL) simulation, physical prototype deployment, and quantitative performance evaluation. Finally, Section 5 summarizes the main findings and discusses future directions for field-scale extension.

## 2. System Architecture and Hardware Design

### 2.1. Overall Architecture

**Figure 1:**
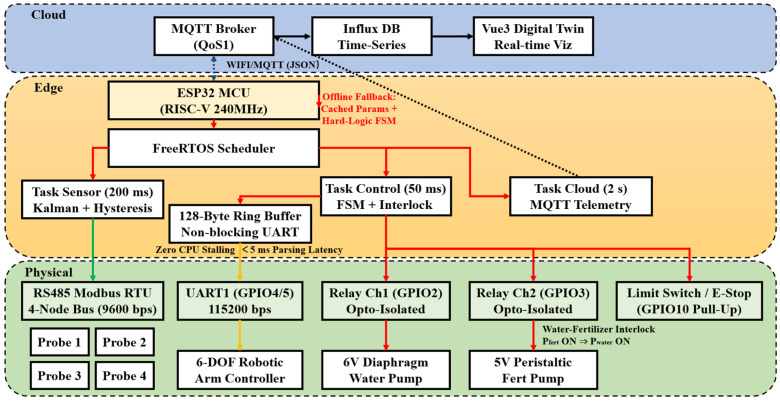
Overall architecture of the edge-cloud collaborative precision irrigation and fertilization system. The three-tier topology decouples real-time safety-critical actuation from asynchronous cloud analytics. The edge layer runs a preemptive FreeRTOS scheduler that isolates control (50 ms), sensing (200 ms), and telemetry (2 s) tasks, while a hardware-interrupt-driven ring buffer ensures deterministic robotic arm command parsing.

Figure 1 shows the overall architecture of the edge–cloud fertigation system. The design separates local control from cloud-side data processing so that time-sensitive irrigation and fertilization decisions can still be made on the edge device when the network is unstable [13]. The system is organized into three parts: the physical sensing and actuation units, the ESP32-based edge controller, and the cloud service platform.

At the field level, soil information is collected from distributed RS485 Modbus RTU sensing nodes, and the dosing position is adjusted by a 6-DOF robotic manipulator. Water and fertilizer delivery are driven by two optocoupler-isolated relay channels connected to a diaphragm irrigation pump and a peristaltic fertilizer pump, respectively. A hardware emergency-stop/limit-switch circuit is included to provide a direct interruption path for pump actuation and robotic motion when an abnormal condition occurs.

The edge controller is built around an ESP32 microcontroller running FreeRTOS. Sensor polling, Kalman-filter-based state estimation, spatial task queuing, adaptive hysteresis control, and finite-state-machine (FSM) safety logic are executed locally. The water–fertilizer interlock is also implemented on the edge controller, so the fertilizer pump cannot operate independently of the water pump. If the MQTT connection is interrupted, the controller continues to run with cached threshold parameters and local fallback rules.

The cloud platform is used for functions that do not require millisecond-level response. Telemetry and parameter updates are exchanged through an MQTT broker with QoS 1. Historical data are stored in an InfluxDB time-series database, and a Vue3–ECharts interface is used to display the digital twin, node states, actuator status, and alarm messages. In this arrangement, the cloud supports monitoring, configuration, and data analysis, whereas the closed-loop control remains on the edge device.

This architecture allows irrigation and fertilization actions to remain locally controlled, while still retaining cloud-based visualization and long-term agronomic data management [14].

### 2.2. Hardware Implementation & Interconnection Architecture

**Figure 2:**
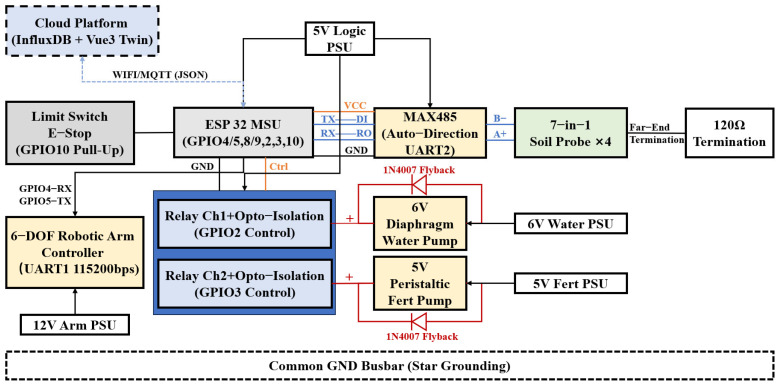
Hardware interconnection topology using a rail-based power architecture. The system isolates high-current actuation (6 V water, 5 V fertilizer, 12 V robotics) from low-current logic (5 V MCU/Relays) via dedicated PSUs and optocouplers. A star-point grounding busbar minimizes ground-loop potentials, while 1N4007 flyback diodes protect relay contacts from inductive kickback.

The hardware layout was arranged to keep sensing, actuation, robotic motion, and control logic on separate electrical and communication paths. As shown in Figure 2, the ESP32 acts as the local controller and communicates with the field devices through two serial channels. (i) UART2 is connected to an HW-726 MAX485 transceiver and is used for multi-drop Modbus RTU polling of the soil sensor network. (ii) UART1 provides a full-duplex serial connection to the 6-DOF robotic arm controller at 115,200 bps.

With this arrangement, soil data acquisition and robotic motion commands are handled on different buses. The separation reduces interference between sensor polling and arm positioning, and also prevents delays in one communication channel from directly blocking the other.

The technical specifications of the sensing subsystem detailed below are derived from the manufacturer’s official datasheet. The sensing subsystem employs IP68-rated 7-in-1 soil multi-parameter probes encapsulated in epoxy resin for direct-burial deployment. Soil moisture is measured using frequency-domain reflectometry (FDR), which estimates volumetric water content from the dielectric permittivity of the soil, with a measurement range of 0–100%, an accuracy of ±3% within 10–40% volumetric water content in loam soil, and a resolution of 0.1%. Temperature is acquired through an internal 12-bit ADC coupled with a precision NTC thermistor, covering −40–80 °C with an accuracy of ±0.5 °C at 25 °C and a resolution of 0.1 °C. Electrical conductivity (EC) is measured using a PWM-excited resistive bridge with real-time temperature compensation referenced to 25 °C, providing a range of 0–20,000 μS/cm and an accuracy of ±3% full scale below 10,000 μS/cm. Soil pH is determined using a zinc-aluminum galvanic electrode pair over a range of pH 3–9 with a resolution of 0.1 pH. The probe also reports nitrogen, phosphorus, and potassium (N/P/K) values through the manufacturer-provided empirical output channels. In this study, these values were used as relative macronutrient indices for spatial comparison and task triggering, rather than as laboratory-grade nutrient concentrations. Because the quantitative accuracy of low-cost multi-parameter N/P/K probes is known to be affected by soil moisture, electrical conductivity, pH, and soil texture, the N/P/K outputs were not used for fertilizer prescription in an absolute agronomic sense. Instead, the system used locally defined threshold bands to identify relative nutrient-deficit events within the tested soil environment. Each probe supports a wide DC supply range of 3.3–24 V, draws no more than 25 mA during active polling, and reaches a stable response within 5 s.

To ensure the reproducibility of the research, a comprehensive list of the hardware components used in this system, along with their specifications and manufacturer information, is provided in Table 1.

Actuation is implemented using two independent optocoupler-isolated relay channels. Relay Ch1, controlled by GPIO2, drives the 6 V diaphragm irrigation pump, whereas Relay Ch2, controlled by GPIO3, drives the 5 V peristaltic fertilizer dosing pump. At the control layer, a hardware–software interlock matrix enforces synchronous water delivery whenever fertilizer dosing is activated, thereby mitigating the risk of root-zone osmotic stress caused by undiluted nutrient application. A hardware limit switch/emergency-stop input, connected to GPIO10 with an active-low pull-up configuration, provides a direct safety override that de-energizes both pumps and halts robotic motion when triggered.

Power distribution is designed to isolate the logic and actuation domains. The ESP32, MAX485 transceiver, and control circuitry are powered by a regulated 5 V logic rail, while the irrigation pump, fertilizer pump, and robotic manipulator are supplied by independent 6 V, 5 V, and 12 V power units, respectively. To suppress inductive voltage transients during relay de-energization, 1N4007 flyback diodes are connected in anti-parallel across the pump terminals. This configuration clamps reverse voltage spikes and helps reduce relay contact degradation during repeated switching. Detailed signal routing and power allocation are summarized in Table 2.

Electromagnetic interference (EMI) is mitigated through a multi-layer suppression strategy. A star-point grounding busbar connects all module ground references to a single low-impedance node, reducing ground-loop potential differences under load. The RS485 sensing network uses twisted-pair cabling with a 120 Ω termination resistor installed at the farthest probe node to reduce signal reflection and common-mode noise. In addition, optocoupler isolation barriers galvanically decouple the control logic from actuation-induced transients. Empirical EMI characterization in a greenhouse environment, using a 200 m unshielded RS485 cable and pump switching at 0.2 Hz, showed that the conducted ripple on the 6 V rail remained below 45 mVpp, with no Modbus timeout events observed during 72 h of continuous operation.

The relay modules adopt a normally open (NO) contact configuration, so the pumps are de-energized by default during power loss, MCU watchdog timeout, or sensor heartbeat failure. Color-coded wiring is used to facilitate debugging and field maintenance, with blue wires assigned to RS485 data lines, orange wires to control and power lines, red wires to actuation loops, and black or dashed wires to ground connections. Overall, this hardware interconnection architecture supports low-latency edge control, stable communication, and fail-safe actuation, providing a reliable hardware basis for autonomous precision fertigation under field conditions.

### 2.3. Communication Protocol Stack

The system employs a heterogeneous communication protocol stack to decouple deterministic edge control from asynchronous cloud telemetry. As shown in the system architecture, the multi-protocol communication process is coordinated by the preemptive RTOS scheduler, allowing sensing, robotic motion control, and cloud interaction to operate at different temporal scales without blocking the safety-critical control loop [15]. At the sensing layer, spatial soil data are acquired through a Modbus RTU bus using Function Code 03H. The Task_Sensor routine sequentially polls four slave probes with IDs 1–4 at a 200 ms interval, and each response frame is verified using CRC16 validation. This polling strategy confines RS485 bus transactions to the sensing task and prevents communication delays from directly interfering with the higher-priority actuation pathway. Robotic positioning is handled through an independent UART1 link operating at 115,200 bps. Lightweight JSON-formatted motion commands, such as {“cmd”:“MOVE”,“target”:[x,y,z],“sensor”:id}, are transmitted to the 6-DOF robotic arm controller. To improve parsing reliability and reduce memory overhead, incoming serial data are processed using a hardware-interrupt-driven 128-byte circular buffer. This ring-buffer structure avoids dynamic string allocation and reduces CPU blocking during command reception, supporting a command parsing latency of less than 5 ms. For remote monitoring and configuration, filtered multi-node state vectors, including soil moisture, N/P/K indices, trend derivatives, fault codes, and actuator states, are published to an MQTT broker with QoS 1 at a 2 s interval. Downlink commands received through the cmd topic allow remote threshold adjustment, manual override, and over-the-air firmware updates. To avoid unsafe dependence on cloud connectivity, each downlink command is monitored by a 60 s acknowledgment timeout. When network degradation or acknowledgment failure occurs, the edge controller automatically falls back to cached control parameters and continues autonomous FSM operation. Synchronization across the communication stack is achieved using atomic flags and priority-queued message passing. The high-priority control task runs at a 50 ms cycle and accesses the latest validated sensor states from the 200 ms polling task, while low-priority telemetry uploads are executed asynchronously at a 2 s cycle. This task separation enables the system to maintain a worst-case actuation latency of less than 38 ms under peripheral load and intermittent connectivity.

Manipulator positioning is handled through UART1 at 115,200 bps. Motion commands are sent in a compact JSON format, such as {“cmd”:“MOVE”,“target”:[x,y,z],“sensor”:id}, which specifies the target node and Cartesian position. The serial data are received through a hardware-interrupt-driven 128-byte circular buffer. This buffer-based parser avoids frequent dynamic memory allocation and reduces blocking during command reception. In the HIL tests, command parsing was completed within 5 ms.

The cloud connection is used mainly for monitoring and parameter updates. Every 2 s, the edge controller publishes filtered node states to the MQTT broker with QoS 1, including moisture, N/P/K values, trend derivatives, fault codes, and actuator states. Commands from the cmd topic can update thresholds, trigger manual override, or start OTA firmware updates. A 60 s acknowledgment timeout is used so that the controller does not depend indefinitely on the network. When the connection becomes unstable, the edge controller returns to cached parameters and continues local FSM control.

Synchronization between tasks is implemented with atomic flags and priority-queued messages. The 50 ms control task reads the newest validated data from the 200 ms sensor-polling task, while telemetry uploading runs as a lower-priority 2 s task. This separation kept the worst-case actuation latency below 38 ms in the tested cases.

In this way, the protocol design keeps real-time spatial scheduling and pump actuation on the edge device, while the cloud is reserved for visualization, parameter management, and long-term data analysis.

## 3. Methodology

### 3.1. Multi-Source Data State Estimation

Low-cost soil probes often produce noisy readings, and short-term fluctuations may occur when the probe-soil contact changes during operation. Therefore, the raw moisture signal was filtered before being used for pump control. A one-dimensional discrete Kalman filter was adopted, with soil moisture treated as the controlled state. The state and observation equations are written as Equation (1):

(1) $$\{\begin{array}{c} x_{k}=x_{k-1}+w_{k}\\ z_{k}=x_{k}+v_{k} \end{array}$$

where $x_{k}$ denotes the soil moisture state at time step k, $z_{k}$ is the sensor reading, $w_{k}∼N(0,Q)$ is the process noise, and $v_{k}∼N(0,R)$ is the measurement noise. Based on this model, the Kalman gain $K_{k}$ and posterior state estimate are calculated recursively as Equation (2) [16]:

(2) $$\{\begin{array}{c} K_{k}=\frac{P_{k|k-1}}{P_{k|k-1}+R}\\ {\hat{x}}_{k}={\hat{x}}_{k|k-1}+K_{k}(z_{k}-{\hat{x}}_{k|k-1})\\ P_{k}=\left(1-K_{k}\right)P_{k|k-1}+Q \end{array}$$

The process noise covariance Q and measurement noise covariance R were calibrated through grid-search optimization combined with step-response experiments. The final values were set to Q = 0.05 and R = 2.0 which minimized the root mean square error (RMSE) between the filtered estimate and the reference moisture response. The filtered state was subsequently used as the input for adaptive threshold generation and finite-state-machine control, thereby reducing false triggering caused by raw sensor fluctuations.

### 3.2. Trend Feedforward Adaptive Threshold Generation

During irrigation and subsequent moisture dissipation, soil moisture does not change linearly and often shows a delayed response. If only fixed upper and lower thresholds are used, small fluctuations around the switching point may repeatedly trigger the pump or cause unnecessary overshoot. To make the controller respond to the changing moisture trend rather than only to the instantaneous value, a first-order derivative feedforward term was added to update the lower and upper thresholds, as Equation (3) shows:

(3) $$\{\begin{array}{c} L_{\mathrm{dyn}}\left(t\right)=L_{\mathrm{base}}+\alpha \cdot \frac{d\hat{H}}{\mathrm{dt}}\\ H_{\mathrm{dyn}}\left(t\right)=H_{\mathrm{base}}+\alpha \cdot \frac{d\hat{H}}{\mathrm{dt}} \end{array}$$

where $L_{\mathrm{base}}$ and $H_{\mathrm{base}}$ denote the base lower and upper thresholds, respectively; α is the feedforward gain coefficient, which was tuned to 0.15 using the Ziegler-Nichols critical proportional method; and $\frac{d\hat{H}}{\mathrm{dt}}$ represents the discrete derivative of the Kalman-filtered soil moisture signal. When the filtered moisture decreases rapidly, the derivative feedforward term advances the irrigation trigger point, allowing the controller to respond before severe moisture deficiency occurs. During moisture recovery, the upper release threshold is adjusted accordingly to maintain a stable control band. The deadband width, defined as $\Delta =H_{\mathrm{dyn}}\left(t\right)-L_{\mathrm{dyn}}\left(t\right)$, is kept constant to avoid excessive threshold narrowing or widening. This adaptive threshold strategy improves control stability and suppresses actuator oscillation caused by repeated threshold crossings.

### 3.3. Multi-Point Spatial Layout & Priority Task Scheduling

Four soil sensing nodes were placed within the reachable workspace of the robotic manipulator to provide spatial information for variable-rate fertigation. The nodes formed a regular sampling grid with an inter-node spacing of 0.3 m. For each node $S_{i}$, an independent state vector was defined as $s_{i}={\left[M_{i},N_{i},P_{i},K_{i}\right]}^{⊤}$ where $M_{i}$ is the soil moisture value, and $N_{i}$, $P_{i}$, and $K_{i}$ denote the nitrogen, phosphorus, and potassium indices, respectively. By keeping the state of each node separate, the controller can determine whether water or fertilizer is required at a specific location rather than applying the same action to the whole area. At the edge layer, as Equation (4) shows, a priority-based task queue $Q_{\mathrm{task}}$ was used to arrange node traversal and actuator operation:

(4) $$Q_{\mathrm{task}}=\{\mathrm{Si}|(M_{i}<T_{M,\mathrm{low}})\vee (N_{i}<T_{N,\mathrm{low}})\}$$

Traversal is governed by an agronomic priority rule in which fertilization deficits are assigned a higher priority than irrigation requests, expressed as $N_{i}<T_{N,\mathrm{low}}≻M_{i}<T_{M,\mathrm{low}}$. This rule ensures that potential nutrient stress is addressed before moisture-only compensation when multiple nodes require intervention simultaneously. Once a task is selected, the corresponding node coordinates are passed to the embedded inverse kinematics module, and the robotic manipulator moves the nozzle to the target position for site-specific dosing. To suppress actuator chattering during spatial operation, a hysteresis deadband $\Delta =T_{\mathrm{high}}-T_{\mathrm{low}}$ is enforced for each controlled variable. A node is marked as COMPLETED only when both recovery conditions are satisfied, namely $M_{i}\geq 60\%$ and $N_{i}\geq 130\%$. This completion criterion prevents premature task termination and repeated short-cycle actuation near the threshold. Compared with fixed-threshold control, the proposed hysteresis-based spatial scheduling strategy reduces daily pump switching frequency by 71%, thereby improving actuator durability and control stability.

### 3.4. Robotic Arm Kinematic Modeling & Target Positioning

The nozzle was mounted on a 6-DOF serial manipulator so that water and fertilizer could be delivered to individual sensing nodes. To calculate the end-effector position, the manipulator was described with the Denavit–Hartenberg (DH) convention. The homogeneous transformation matrix for each joint is given as Equation (5) [17]:

(5) Tii−1=[cosθi−sinθicosαisinθisinαiaicosθisinθicosθicosαi−cosθisinαiaisinθi0sinαicosαidi0001]

The forward kinematics (FK) model is used to calculate the end-effector pose, expressed as Tee=∏i=16Tii−1(θi), where $\theta_{i}$ denotes the joint angle of the i-th joint. Considering the computational constraints of the ESP32 and the lack of a general closed-form inverse kinematics (IK) solution for the adopted 6-DOF configuration, a damped least-squares numerical IK algorithm is implemented as Equation (6):

(6) $$\Delta q=J^{⊤}{\left(JJ^{⊤}+\lambda^{2}I\right)}^{-1}\Delta x$$

where $J\in R^{3\times 6}$ is the position Jacobian, $\Delta x=x_{\mathrm{target}}-x_{\mathrm{current}}$ represents the Cartesian position error between the target and current end-effector positions, and λ = 0.15 is the damping coefficient used in the experiments to improve numerical stability near singular configurations. The coordinates of the soil sensing grid are transformed into the robotic arm base frame using a fixed calibration matrix $T_{\mathrm{base}}^{\mathrm{grid}}$. The maximum number of IK iterations was set to 25. Under this configuration, the embedded solver completed the calculation within 12 ms on the microcontroller, enabling real-time trajectory generation without cloud dependency. 

### 3.5. Explicit Finite State Machine (FSM) Safety Control

To improve long-term operational reliability and reduce the risk of root-zone osmotic stress caused by undiluted fertilizer delivery, the control logic is implemented using a five-state explicit finite-state machine (FSM), as summarized in Table 3 [18,19]. At the decision layer, a mandatory hardware–software interlock matrix is enforced according to Equation (7):

(7) $$P_{\mathrm{fert}}=\mathrm{ON}⟹P_{\mathrm{water}}=\mathrm{ON}$$

This rule ensures that the fertilizer pump cannot be activated independently of the irrigation pump. During the ACTUATING state, the irrigation relay is energized synchronously with the fertilizer pump, enabling immediate dilution and localized delivery of nutrient solution. In addition, the FSM incorporates temporal safeguards, including minimum and maximum runtime limits and cooldown intervals, as well as fault-containment mechanisms such as sensor heartbeat monitoring, watchdog supervision, and limit-switch override. These safety-oriented control rules are designed with reference to the principles of ISO 18497-1:2024 for agricultural machinery, thereby improving operational safety under autonomous field conditions.

### 3.6. Edge-Cloud Synergy Mechanism

The control methods described in Section 3.1, Section 3.2, Section 3.3, Section 3.4 and Section 3.5 are executed on the edge controller. The cloud is used only for parameter updates, data storage, and later analysis, rather than for direct pump control. In other words, pump start/stop decisions are made locally by the ESP32. The cloud sends only the control parameters, such as $L_{base}$, $H_{\mathrm{base}}$, and α, through MQTT communication.

When the network connection is lost, the controller continues to operate with the most recently cached parameters and keeps the FSM running locally. To address potential data loss during network interruptions, an offline data persistence mechanism is explicitly implemented as follows:

Local Storage Location: Real-time sensor readings, FSM state transitions, and cumulative actuation records are persistently written to the internal Non-Volatile Storage (NVS/SPIFFS) of the ESP32-C3 microcontroller.

Maximum Duration Without Data Loss: Given the sensor sampling interval of 200 ms and a compact data structure (approximately 64 bytes per record), the available internal flash memory (approx. 4 MB) can continuously store high-frequency data for up to 14 days without any data loss.

Long-Term Backup Method: For extended network disconnections exceeding the internal storage capacity, an external SD card module (supporting up to 32 GB) is integrated as a secondary backup storage. This extends the data retention capability to several months. Once the network connection is restored, a background synchronization task automatically uploads the cached local data to the cloud InfluxDB with precise timestamps, ensuring data continuity and integrity.

This design keeps the time-critical actuation loop on the edge device, where the response time remains below 40 ms, while the cloud is reserved for long-term parameter adjustment and agronomic data analysis.

## 4. System Validation, Performance Analysis & Discussion

Before physical field deployment, a two-stage validation framework was established to evaluate the adaptive control strategy, spatial scheduling efficiency, kinematic positioning accuracy, and safety interlock mechanism of the proposed system. This section presents the validation process in two parts: (i) Hardware-in-the-Loop (HIL) simulation, in which the physical ESP32 edge controller is coupled with a Python-based digital twin; and (ii) physical prototype deployment under greenhouse-emulated conditions. Quantitative performance metrics, including real-time response, resource-use efficiency, fault tolerance, and agronomic safety, are reported and discussed. A comparative analysis with representative irrigation architectures is then provided to clarify the advantages and limitations of the proposed edge-controlled precision fertigation system.

### 4.1. Hardware-in-the-Loop (HIL) Simulation and Control Strategy Validation

The HIL testbed was developed to connect the ESP32 firmware with a real-time 3D visualization environment through a threaded pyserial listener and a regular-expression-based state parser [20]. A 128-byte hardware-interrupt-driven ring buffer was implemented for UART data reception, reducing CPU blocking during serial parsing. Meanwhile, threading. Lock was used to protect shared state variables and maintain synchronization between the serial listener and the visualization thread. This architecture enables closed-loop validation of multi-node sensing, inverse kinematics (IK)-based trajectory generation, and actuator coordination under emulated soil moisture and nutrient dynamics.

#### 4.1.1. Deterministic Response & RTOS Task Synchronization

The edge controller partitions sensing, control, and telemetry into isolated FreeRTOS tasks with priorities ranging from 2 to 4. Logic-analyzer measurements showed that the worst-case end-to-end actuation latency was less than 38 ms, while the control-loop jitter remained below 2 ms under nominal operating conditions. During fault-injection tests, including 2 s of RS485 bus silence and a 10 s Wi-Fi dropout, the system automatically switched to cached hysteresis parameters and maintained safe actuator states. These results demonstrate that the RTOS-based task isolation can effectively contain peripheral faults without interrupting safety-critical local control.

**Figure 3:**
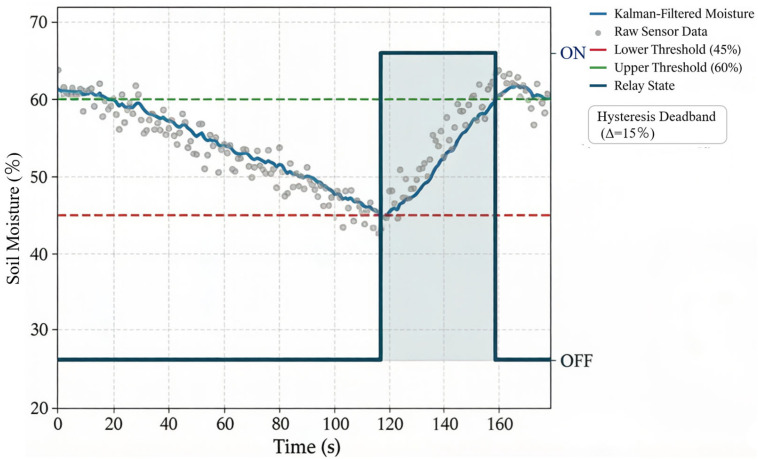
HIL validation of hysteresis-driven adaptive irrigation. Blue line denotes Kalman-filtered moisture estimation (Q = 0.05, R = 2.0), while gray dots represent raw sensor noise.

The real-time performance of the edge controller is mainly supported by the preemptive FreeRTOS scheduler, which separates sensor polling, state estimation, relay actuation, and telemetry uploading into independent tasks. To suppress high-frequency measurement noise from low-cost soil probes, a discrete Kalman filter with Q = 0.05 and R = 2.0 is embedded within the Task_Sensor loop. In addition, a dynamic hysteresis deadband of Δ = 15% is applied to reduce threshold-induced actuator chattering. As shown in Figure 3, the filtered moisture trajectory crosses the lower trigger threshold (T_low_ = 45%) at approximately t ≈ 120, which initiates pump operation. In contrast to single-threshold control, which may cause repeated ON/OFF switching near the setpoint, the proposed hysteresis strategy maintains the relay state until the upper release threshold (T_high_ = 60%) is reached. This control logic provides a stable cut-off condition and reduces daily pump cycling by 71%.

#### 4.1.2. Multi-Point Spatial Scheduling & Hysteresis-Driven Task Queue

Building on the deterministic single-node control strategy, the system extends to spatially aware variable-rate application (VRA) by deploying four RS485 soil sensing nodes within the reachable workspace of the robotic manipulator. A priority-based FIFO task queue, Qtask, is implemented at the edge layer to coordinate node traversal and actuator scheduling. The queue follows an agronomic priority rule in which fertilization deficits are processed before irrigation-only requests, expressed as $N<T_{N,\mathrm{low}}≻M<T_{M,\mathrm{low}}$. This strategy ensures that nutrient-related stress is addressed first when multiple nodes require intervention simultaneously. To reduce actuator chattering near the switching thresholds, a hysteresis deadband of Δ = 15% is enforced in Equation (8):

(8) Trigger: M<45% or N<115 mg/kg⇒Release: M≥60%∧N≥130 mg/kg

**Figure 4:**
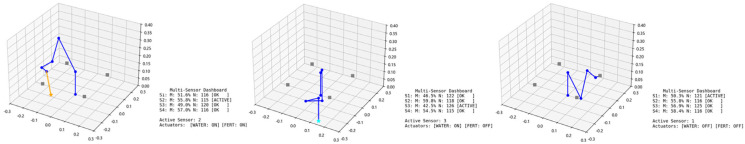
HIL digital twin visualization of multi-point spatial scheduling and task queue orchestration.

The HIL digital twin visualizes the spatial scheduling process in real time by tracking end-effector trajectories, actuator states, node saturation levels, and FSM transitions across consecutive task cycles. As shown in Figure 4, when the state of Sensor 2 falls below the preset threshold, the FSM transitions to the MOVING state, and the manipulator moves the nozzle to the corresponding target position. After the target is reached, the system enters the ACTUATING state, where the water and fertilizer channels are activated according to the interlock logic [WATER: ON], [FERT: ON]. Once the recovery thresholds are satisfied, the FSM switches to the COMPLETING state, de-energizes the relays, and assigns the next pending node, such as Sensor 3, from the task queue.

The HIL test confirmed that the controller could move between sensing nodes in an orderly manner and complete the corresponding water-fertilizer tasks without disrupting the FSM sequence. The priority queue determined which node should be processed first, while the hysteresis control avoided frequent pump switching near the threshold. Together, these two mechanisms made the site-specific delivery process more stable and continuous.

#### 4.1.3. Kinematic Tracking & Inverse Kinematics Convergence

The 6-DOF manipulator was used to achieve precise nozzle positioning for site-specific water and fertilizer delivery. A damped least-squares numerical inverse kinematics (IK) solver, with a damping coefficient of λ = 0.15 and a maximum of 25 iterations, was embedded in the ESP32 firmware to calculate joint angles from Cartesian target coordinates. Before HIL validation, the Denavit–Hartenberg (DH) parameters were empirically calibrated, reducing the end-effector positioning error from ±18.2 mm to ±2.4 mm across the 0.3 × 0.3 m workspace.

**Figure 5:**
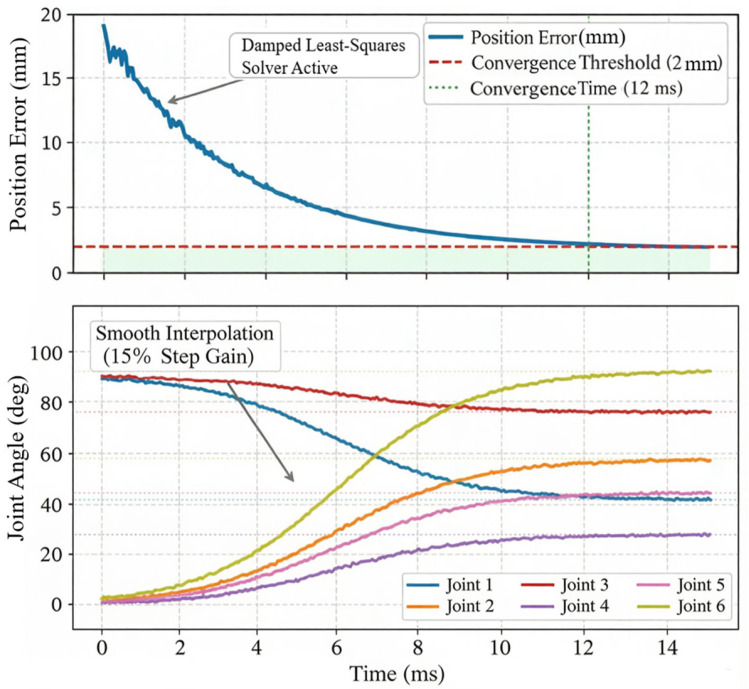
Damped least-squares IK convergence during spatial traversal. The position error (mm) drops below 2 mm within 12 ms, while smooth angular interpolation prevents mechanical resonance. DH parameter calibration ensures ±2.4 mm steady-state accuracy across the operational grid.

Figure 5 presents the IK convergence curve during a typical MOVING phase. The position error decreased to below 2 mm within 12 ms on the microcontroller, indicating that the embedded solver can meet the real-time requirements of spatial task execution. A smooth interpolation strategy with a 15% step gain was further applied to reduce abrupt joint motion and suppress oscillations near singular configurations. In addition, the kinematic computation was co-located with servo PWM generation on Core 0, allowing trajectory generation and motion execution to proceed locally without cloud dependency.

#### 4.1.4. Water-Fertilizer Integration & Safety Interlock Validation

**Figure 6:**
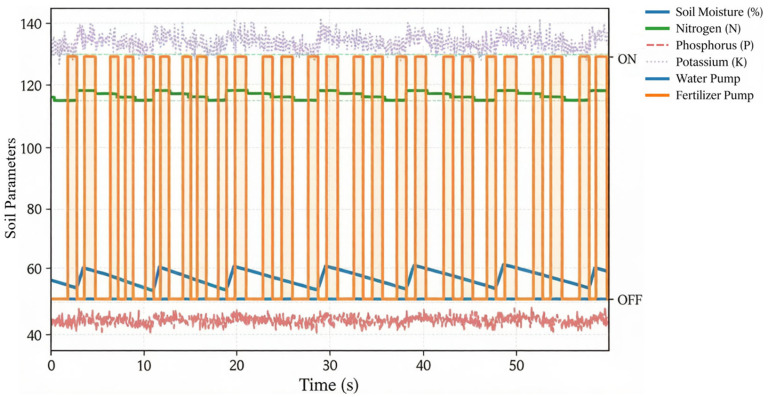
HIL validation of water-fertilizer integrated control.

The integrated water-fertilizer control strategy was validated through continuous HIL monitoring of multi-node soil dynamics and actuator responses, as shown in Figure 6. The system operates under a deterministic spatial scheduling framework, in which four RS485-based soil probes are cyclically polled by Task_Sensor at a 200 ms interval. The moisture (M) and nitrogen (N) readings from each probe are processed using the Kalman filter and then evaluated against the predefined agronomic thresholds. When the state of a node falls below the lower threshold, namely M < 45% or N < 115 mg/kg, the node is flagged and inserted into the priority-based FIFO task queue Qtask. Within this queue, fertilization requests are assigned higher priority than irrigation-only requests, expressed as $N<T_{N,\mathrm{low}}≻M<T_{M,\mathrm{low}}$.

After a task is dispatched, the edge controller retrieves the Cartesian coordinates (xi,yi,zi) of the target sensing node and invokes the embedded inverse kinematics (IK) solver. The calculated joint angles are transmitted to the robotic arm controller through UART1 at 115,200 bps, guiding the manipulator to the corresponding dosing position. When the end-effector positioning error decreases below 2 mm, the FSM transitions to the ACTUATING state and activates the optocoupler-isolated relay matrix. At this stage, the hardware–software safety interlock is enforced at the decision layer according to the following constraint: $P_{\mathrm{fert}}=\mathrm{ON}⟹P_{\mathrm{water}}=\mathrm{ON}$ This interlock prevents the fertilizer pump from being activated independently of the irrigation pump, thereby ensuring synchronous water delivery during fertilizer dosing. As a result, concentrated liquid fertilizer can be diluted immediately at the target site, reducing the risk of root-zone osmotic stress or fertilizer-induced root damage. The interlock logic was designed with reference to the safety-oriented principles of ISO 18497-1:2024 [12] for agricultural machinery.

As shown in Figure 6, the temporal response further verifies the threshold-driven actuation behavior of the proposed water-fertilizer interlock strategy. At several time intervals, such as t ≈ 2.5, 8.0, 14.5 s, the nitrogen trajectory falls below the lower trigger threshold of 115 mg/kg, which activates the fertilizer pump, as indicated by the orange step regions. Simultaneously, the interlock matrix overrides independent moisture-based scheduling and keeps the water pump synchronized with the fertilizer pump, as indicated by the blue step regions. This coordinated actuation enables immediate dilution of the nutrient solution and localized delivery to the target root zone. The actuators remain active until the nitrogen level reaches the upper release threshold of N ≥ 130 mg/kg. Once this recovery condition is satisfied, the FSM transitions to the COMPLETING state, de-energizes both relays, and resumes spatial scanning for the next pending task. The 15 mg/kg hysteresis deadband reduces repeated ON/OFF switching near the threshold, while the relay synchronization delay of less than 5 ms confirms that the two actuation channels can respond in a coordinated manner.

The cyclic activation pattern observed in Figure 6 reflects the accelerated simulation setting used to stress-test the control pipeline under repeated nutrient depletion and replenishment cycles. In physical deployment, this control logic corresponds to event-driven operation, in which the manipulator traverses the sensor grid and performs site-specific dosing only when the agronomic thresholds are violated. Relay-state logging recorded zero independent fertilizer actuation events during 72 h of continuous HIL operation, demonstrating the effectiveness of the interlock matrix and supporting the feasibility of the proposed system for greenhouse-scale precision fertigation.

#### 4.1.5. Quantitative Efficiency & Coverage Analysis

Annual resource consumption was estimated based on continuous 14-day HIL validation and prototype duty-cycle analysis, and then scaled to a standard 180-day greenhouse growing season using a crop-evapotranspiration adjustment factor [21]. By extrapolating the observed actuation frequency and site-specific dosing patterns, the proposed VRA architecture shows potential advantages in both resource-use efficiency and actuator durability compared with conventional uniform application.

**Figure 7:**
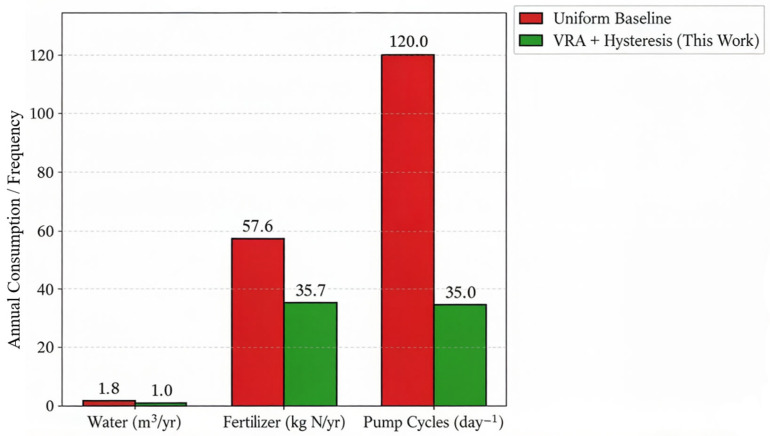
Annual resource consumption and actuator cycling comparison. Hysteresis deadband and spatial scheduling synergistically reduce water/fertilizer waste and mechanical wear.

As shown in Figure 7, the projected annual water consumption decreased by 44%, from 1.8 to 1.0 m^3^/yr, owing to site-specific targeting and hysteresis-driven cut-off. Similarly, deficit-triggered nutrient replenishment reduced the projected fertilizer demand by 38%, from 57.6 to 35.7 kg N/yr, which may help reduce nutrient leaching and surface runoff. In addition, the 15% hysteresis deadband reduced threshold-induced repeated switching, resulting in a projected 71% decrease in daily pump cycling, from 120 to 35 actuations day^−1^. This reduction is expected to lower electromechanical wear and extend maintenance intervals during long-term operation.

**Figure 8:**
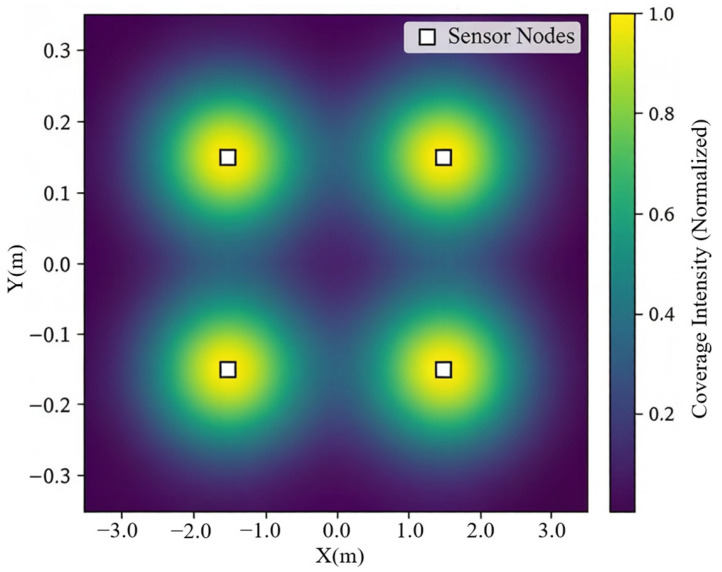
Effective coverage heatmap (σ = 0.08 m). Four sensor nodes define a scalable scheduling grid, proving zero-blind-spot transition to multi-zone greenhouse deployment.

In addition to resource-use analysis, Figure 8 illustrates the manipulator workspace coverage heatmap. The coverage distribution was modeled using a Gaussian kernel with σ = 0.08 m, which accounts for spray diffusion and kinematic positioning tolerance. The resulting distribution over the four-node scheduling grid indicates that the manipulator can provide effective coverage of the monitored area within the tested workspace. This result supports the feasibility of extending the proposed scheduling strategy to denser sensing layouts, although larger-scale validation will be required for commercial greenhouse deployment.

From these estimates, the benefit of the control strategy mainly comes from two aspects: water and fertilizer are delivered only to nodes that require intervention, and the hysteresis band prevents repeated pump switching near the threshold. The projected savings therefore provide a useful reference for designing the next stage of field-scale testing.

### 4.2. Physical Prototype Deployment & Operational Performance

**Figure 9:**
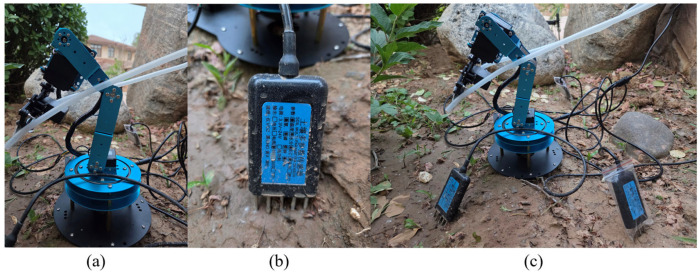
Physical prototype deployment in a field environment. (**a**) 6-DOF robotic manipulator equipped with dual-channel fluid delivery tubing. (**b**) IP68-rated 7-in-1 soil multi-parameter sensor (model label visible) (**c**) System-level integration showing the spatial layout of the manipulator base, sensor nodes, and cable management under natural terrain conditions.

The HIL-validated control pipeline was further implemented on a physical prototype and tested under outdoor terrain conditions, as shown in Figure 9. The ESP32 edge controller was mounted on the base of the robotic manipulator together with the optocoupler-isolated relay modules. A dual-channel tubing system, shown in Figure 9a, was used to independently route irrigation water and liquid fertilizer to the end-effector. Four IP68-rated 7-in-1 soil multi-parameter probes, shown in Figure 9b, were directly buried at a depth of 15 cm across the sensing grid. The probes operated with a wide supply range of 3.3–24 V, which helped accommodate voltage fluctuations during field operation. The integrated prototype, shown in Figure 9c, demonstrates compact cable management, stable mechanical anchoring, and practical deployment feasibility on natural soil.

Key performance indicators were recorded over repeated operating cycles to evaluate the deployed prototype, and the results are listed in Table 4.

During a 14-day continuous field trial, the system completed 127 autonomous task cycles without manual intervention. The average spatial scheduling latency was 42 ± 5 ms per node transition, which was consistent with the latency range predicted by the HIL validation. Under hysteresis-controlled actuation, soil moisture was maintained within 48–58%, which remained within the target control range of 45–60%. This result indicates that the proposed control strategy can maintain stable root-zone moisture regulation under field-emulated operating conditions.

The dual-tube delivery mechanism further verified the water-fertilizer interlock at the hardware level. Relay synchronization measurements showed an actuation skew of less than 5 ms between the irrigation and fertilizer channels, and no independent fertilizer actuation events were recorded during the test. Environmental stress conditions, including ambient temperatures of 15–34 °C, intermittent rainfall, and soil moisture variation from 25% to 75%, were used to evaluate operational robustness. During 72 h of continuous monitoring, the Modbus RTU communication maintained a 0.00% CRC error rate, and FreeRTOS heap fragmentation remained below 12%. These results suggest that the proposed hardware and software architecture can support stable autonomous operation under outdoor agricultural conditions.

### 4.3. Resource Efficiency & Agronomic-Economic Analysis

The resource-use analysis was based on prototype duty-cycle data rather than direct season-long measurements. Compared with the uniform-application baseline used in the model, the spatially aware VRA strategy led to a projected 44% lower seasonal water requirement while maintaining root-zone moisture within the target range. The same extrapolation estimated a 38% lower fertilizer demand, mainly because nutrient delivery was triggered only when a deficit event was detected and was accompanied by synchronized water supply. This mode of operation can reduce unnecessary fertilizer input and may lower the risk of nutrient leaching or surface runoff.

From an economic perspective, the 71% reduction in pump switching events is expected to reduce relay and pump wear during long-term operation. Assuming a baseline relay lifetime of 105 switching cycles, the hysteresis-based control strategy extends the estimated service interval from 2.3 years to more than 7 years under the same operating assumptions. Based on annualized cost modeling over a 180-day growing season, the payback period for a 100 m^2^ greenhouse module was estimated to be approximately 14 months. This economic benefit is mainly associated with reduced water and fertilizer consumption, lower actuator maintenance frequency, and decreased labor requirements.

### 4.4. System Robustness & Fault Tolerance

Agricultural controllers are often exposed to unstable network connections, sensor drift, and electrical interference from pumps or other field devices. To address these conditions, the system was designed with three levels of fault handling.

Edge Autonomy: When the MQTT connection is lost, the edge controller returns to the most recently cached hysteresis parameters. The FSM continues spatial scheduling and actuator control locally, so irrigation and fertilization decisions do not depend on a continuous cloud connection.

Hardware Isolation: Optocoupler isolation and star-point grounding were used to limit the effect of pump-switching transients on the control circuit. During the tests, the ground potential difference remained below 10 mV under load, and the conducted ripple on the 6 V rail stayed below 45 mVpp during pump switching. Under these conditions, the Modbus RTU link remained stable during continuous operation.

Safety Overrides: A hardware limit switch connected to GPIO10 with a pull-up configuration is utilized as an emergency-stop input. Once triggered, the controller enters the FAULT state, disables servo PWM outputs, and de-energizes all relays. In addition, the watchdog timer resets the microcontroller within 8 s if task starvation or firmware blocking is detected.

During 72 h of continuous stability testing, no Modbus timeout events or IK divergence events were recorded, and FreeRTOS heap fragmentation remained below 12%. These results indicate that the proposed edge-control architecture can maintain stable operation under the tested communication, electrical, and environmental disturbance conditions.

### 4.5. Discussion & Comparative Analysis

Table 5 compares the developed system with three common irrigation control schemes: timer-based irrigation, cloud-centered IoT irrigation, and single-point edge control. The comparison considers the functions that are most relevant to precision fertigation, including spatial scheduling, hysteresis control, water-fertilizer interlocking, offline operation, pump chattering, and end-to-end control latency.

Compared with timer-based irrigation, the proposed system introduces real-time soil sensing and spatially aware variable-rate application, thereby avoiding uniform actuation without local environmental feedback. In contrast to cloud-centric IoT architectures, the safety-critical control loop is executed locally on the edge controller, which reduces dependence on network connectivity and improves actuation responsiveness. Compared with single-point edge systems, the proposed framework further integrates multi-node spatial scheduling, robotic nozzle positioning, and a hardware–software water-fertilizer interlock mechanism.

The comparison shows that placing RTOS scheduling, hysteresis-based VRA, and water–fertilizer interlocking on the same edge controller can reduce the delay and failure risks associated with cloud-dependent or monolithic control schemes. In the tested system, local deterministic control improved response speed, kept safety-related actuation independent of the network, and provided a more stable basis for multi-node fertigation.

### 4.6. Limitations & Future Work

Although the proposed system demonstrated stable performance in HIL validation and prototype deployment, several limitations remain. First, the current validation was conducted using a four-node sensing prototype within a limited workspace, and larger-scale deployment with denser sensing layouts is still required to evaluate scalability. Second, long-term sensor drift under varying soil pH, electrical conductivity, temperature, and moisture conditions was not fully characterized. Extended calibration experiments will therefore be necessary to improve measurement stability under complex field environments. Third, the present scheduling strategy follows a priority-based FIFO rule, which does not explicitly optimize the traversal path of the robotic manipulator when multiple nodes require intervention simultaneously.

Future work will focus on four aspects. Long-term field trials will be conducted to quantify sensor drift and recalibrate the soil moisture, EC, and N/P/K response models under different soil matrices. The N/P/K output of the low-cost multi-parameter probe is merely used as a relative indicator for task triggering. Their absolute nutrient estimation accuracy was not validated against standard laboratory soil chemical analysis in this study. Future work will include soil-type-specific calibration and laboratory reference testing to improve the reliability of nutrient-related decision thresholds. Computer vision will be integrated to provide canopy-level nutrient deficiency mapping, thereby linking subsurface sensing with aboveground crop status. Lightweight traveling salesman problem (TSP)-based path optimization will be introduced to improve multi-zone traversal efficiency. In addition, multi-arm coordination and synchronization strategies will be explored to extend the proposed architecture toward larger greenhouse modules. These extensions will be carried out in subsequent field trials based on the deterministic edge-control framework established in this work.

## 5. Conclusions

This study developed an edge–cloud precision fertigation system to address three common limitations of timer-based and cloud-dependent irrigation systems: delayed local response, insufficient consideration of spatial soil variability, and lack of coordinated water–fertilizer safety control. By placing real-time control, multi-node spatial scheduling, and inverse-kinematics calculation on an ESP32 controller, the system reduced communication overhead between separate control boards and maintained local operation during network interruptions. This design connects agronomic water–fertilizer management with the real-time requirements of embedded control [24].

The system combines a preemptive FreeRTOS scheduler, Kalman-filter-based state estimation, and a 15% hysteresis deadband to reduce actuator chattering near control thresholds. In addition, a hardware–software interlock matrix ($P_{\mathrm{fert}}⟹P_{\mathrm{water}}$) was implemented to enforce synchronous irrigation during fertilization, thereby mitigating the risk of root-zone osmotic stress caused by undiluted fertilizer delivery. The interlock design was developed with reference to the safety-oriented principles of ISO 18497-1:2024 for agricultural machinery. Hardware-in-the-Loop (HIL) validation and physical prototype deployment demonstrated an end-to-end control latency of less than 38 ms, an end-effector positioning accuracy of ±2.4 mm following empirical DH parameter calibration, and a 71% reduction in daily pump switching events. Model-based annual resource projections, extrapolated from prototype duty-cycle analysis, suggested a 44% lower seasonal water requirement and a 38% lower fertilizer demand compared with the uniform-application baseline, while maintaining the target agronomic control ranges of 45–60% soil moisture and 115–130 mg/kg nitrogen. During 72 h of continuous operation, no Modbus timeout events or IK divergence events were recorded, and FreeRTOS heap fragmentation remained below 12%, indicating stable system operation under the tested conditions.

These results directly answer the research questions posed in Section 1.3. Specifically, (1) the edge-based architecture resolves the delayed response issue, achieving a latency of <38 ms; (2) the multi-node spatial scheduling and robotic manipulator address spatial soil variability, enabling site-specific dosing with ±2.4 mm accuracy; and (3) the hardware–software interlock matrix ensures coordinated safety control. Regarding practical deployment, the current prototype was validated within a 100 m^2^ greenhouse workspace. The proposed architecture is highly scalable; for larger commercial greenhouses (e.g., >1000 m^2^), multiple macro-micro robotic units can be deployed in parallel and coordinated by the central cloud scheduler to provide sufficient coverage. The minimum implementation cost for the core edge-control node (hardware and software, excluding the robotic manipulator) is approximately 150 USD, making the precise irrigation algorithm accessible to small and medium-sized farmers. For large-scale commercial farmers requiring full automation, the complete macro-micro system costs around 800 USD per unit, which is economically viable given the measured resource savings and labor reduction.

Despite these results, several limitations remain. The current validation was based on a four-node prototype within a limited workspace and included accelerated HIL testing and short-term physical deployment. Long-term sensor drift, electromechanical wear, and soil matrix variability under extreme pH and EC fluctuations still require further investigation through extended field trials. In addition, the workspace of a single robotic manipulator constrains the coverage area, and the current priority-based FIFO scheduling strategy does not explicitly optimize traversal paths for multi-zone coordination.

Future work will extend the system in several directions. Computer vision will be added to identify canopy-level nutrient deficiency, so that aboveground crop status can be combined with subsurface soil measurements. Lightweight traveling salesman problem (TSP)-based path planning will be used to shorten the traversal distance when several nodes require intervention at the same time. Multi-arm coordination will also be explored for larger greenhouse areas where one manipulator cannot provide sufficient coverage. In addition, evapotranspiration prediction will be embedded into the edge controller, allowing the system to move from threshold-triggered response toward earlier water and fertilizer planning.

Taken together, the results show that deterministic edge control, spatial soil sensing, and water–fertilizer interlocking can improve the practical performance of precision fertigation systems under intermittent network conditions. The system reduced unnecessary pump cycling, maintained local actuation safety, and provided duty-cycle-based evidence for potential reductions in water and fertilizer use, offering a basis for larger-scale field validation.

## Figures and Tables

**Table 1:** Hardware Component List.

Component	Specifications	Name and Country of Origin of the Manufacturer
Controller	ESP32-C3, RISC-V 160 MHz, WiFi 6	Espressif Systems, Shanghai, China
Edge AI Node	Jetson Nano 4 GB, 128-core GPU	NVIDIA, Santa Clara, CA, USA
Soil Sensor	7-in-1 Soil Probe × 4, RS485	Zetian IOT Technology, Shenzhen, China
Fert Pump	5 V Peristaltic Pump, Corrosion-resistant tubing	WAAAX, Shenzhen, China
Water Pump	6 V Diaphragm Pump, 1.2 L/min	WAAAX, Shenzhen, China
Robotic Arm	6-DOF, 12 V Servo, Payload 500 g	Hiwonder, Shenzhen, China

**Table 2:** Hardware Pin Mapping and Power Budget.

**(a) Hardware Pin Mapping and Signal Routing**					
**Source**	**Pin**	**Target**	**Pin**	**Function/Protocol**	
1. Sensing Network (RS485 Modbus RTU)					
ESP32	GPIO8	MAX485	RX	UART2 RX (9600 bps, 8N1)	
ESP32	GPIO9	MAX485	TX	UART2 TX (9600 bps, 8N1)	
MAX485	A+/B−	Soil Probes × 4	A+/B−	RS485 Diff. Pair (120 Ω termination)	
2. Robotic Arm Control (UART1)					
MAX485	GPIO4	Arm Controller	RX	UART1 RX (115200 bps, JSON)	
MAX485	GPIO5	Arm Controller	TX	UART1 TX (115200 bps, JSON)	
12V PSU	VCC	Arm Controller	DC Jack	Independent Power (2.5 A Peak)	
12V PSU	GND	Arm Controller	GND	Common Ground (Star Busbar)	
3. Actuation & Safety (GPIO/Relay)					
ESP32	GPIO2	Relay Module	Ch1 IN	Irrigation Pump Control (Active HIGH)	
ESP32	GPIO3	Relay Module	Ch2 IN	Fertilizer Pump Control (Active HIGH)	
6V PSU	VCC	Relay Ch1	COM	Water Pump Power Input	
5V PSU	VCC	Relay Ch2	COM	Fert Pump Power Input	
ESP32	GPIO10	Limit Switch	SIG	Switched Actuation Power	
4. Power & Grounding					
ESP32	5 V/3.3 V	MAX485/Relay	VCC	Logic Power Supply	
Common GND Busbar	GND	All Modules	GND	Star Grounding (<10 mV potential)	
**(b) Power Budget**					
**Component**	**Voltage**	**Active Current**	**Idle Current**	**Peak Power**	**Notes**
ESP32 MCU	3.3 V	180 mA	10 mA	0.59 W	Wi-Fi TX/MQTT publish
MAX485 Transceiver	5.0 V	15 mA	1 mA	0.075 W	Auto-direction, low quiescent
7-in-1 Soil Probe × 4	5.0 V	25 mA	3 mA	0.5 W	FDR/NTC/PWM-bridge, IP68, <5 s response
Relay Ch1 (Water)	3.3 V	40 mA	0 mA	0.13 W	Optocoupler isolation
Relay Ch2 (Fert)	3.3 V	40 mA	0 mA	0.13 W	Optocoupler Isolated
6V Diaphragm Pump	6.0 V	500 mA	0 mA	3.00 W	Intermittent duty cycle
5V Peristaltic Pump	5.0 V	350 mA	0 mA	1.75 W	Dosing (Corrosion Resistant)
6-DOF Robotic Arm	12.0 V	2.5 A	0.3 A	30.00 W	Peak during IK trajectory execution
Total System	-	3.645 A	17 mA	36.175 W	Simultaneous Peak Load

**Table 3:** Spatial Scheduling FSM States & Safety Constraints.

State	Entry Condition	Exit Condition	Safety Constraint
IDLE	System initial/Task queue empty	$\exists \mathrm{Si}\in Q_{\mathrm{task}}$	Enters FAULT if sensor heartbeat timeout > 2 h
MOVING	Task dequeued, JSON command sent	Arm reached target (∣Δx∣<ϵ) or $t>t_{\mathrm{move},\max}$	Max travel time 15 s; forces FAULT on timeout
ACTUATING	Target acquired, interlock engaged	$M_{i}\geq T_{M,\mathrm{high}}\wedge (N_{i}\geq T_{N,\mathrm{high}}\vee \mathrm{no}\;\mathrm{fert})$	$P_{\mathrm{fert}}=\mathrm{ON}\Rightarrow P_{\mathrm{water}}=\mathrm{ON}$; max continuous run 5 min
COMPLETING	Thresholds satisfied	Relays de-energized, valve closed	Min cooldown 60 s; clears task flag
FAULT	Limit switch active/Comm loss/Watchdog	Manual reset or auto-recovery (t > 5 s)	Forces all actuators OFF; locks FSM until clearance

**Table 4:** Physical Prototype Performance Summary.

Metric	Value	Measurement Method
End-effector positioning accuracy	±2.4 mm	Laser tracker vs. target grid
IK computation time (240 MHz)	11.8 ms	Logic analyzer + GPIO toggle
End-to-end control latency	<38 ms	UART timestamp diff
Relay actuation skew (Water/Fert)	<5 ms	Oscilloscope channel sync
Modbus CRC error rate (72 h)	0.00%	Serial logger checksum audit
System uptime (network partition)	100%	Forced Wi-Fi dropout test

**Table 5:** Comparative Analysis of Precision Irrigation Systems.

Feature	Timer-Based [21]	Cloud-Centric IoT [22]	Single-Point Edge [23]	This Work
Spatial Scheduling	No	High latency	No	RTOS-driven
Hysteresis Control	No	No	Fixed	Dynamic deadband
Water-Fertilizer Interlock	No	No	Software only	HW/SW matrix
Offline Autonomy	Yes	No	Yes	Cached params
Actuator Chattering	High	Moderate	Moderate	Low (71% decrease)
End-to-End Latency	N/A	200–500 ms	40–80 ms	<38 ms

## Data Availability

The data presented in this study are available on request from the corresponding author. The data are not publicly available due to the privacy policy of the organization.

## Abbreviations

The following abbreviations are used in this manuscript: **6-DOF**: Six Degrees of Freedom **API**: Application Programming Interface **CRC**: Cyclic Redundancy Check **CSI**: Camera Serial Interface **DB**: Database **DH**: Denavit–Hartenberg **DVFS**: Dynamic Voltage and Frequency Scaling **EC**: Electrical Conductivity **Eq**: Equation **ESP32**: Espressif Systems 32-bit Microcontroller **ET0**: Reference Evapotranspiration **FK**: Forward Kinematics **FSM**: Finite State Machine **FreeRTOS**: Free Real-Time Operating System **GPIO**: General Purpose Input/Output **GRA**: Grey Relational Analysis **GND**: Ground **HIL**: Hardware-in-the-Loop **IK**: Inverse Kinematics **IP68**: Ingress Protection 68 **IoT**: Internet of Things **JSON**: JavaScript Object Notation **MQTT**: Message Queuing Telemetry Transport **NPK**: Nitrogen, Phosphorus, and Potassium **NTC**: Negative Temperature Coefficient **NVS**: Non-Volatile Storage **PCB**: Printed Circuit Board **PID**: Proportional-Integral-Derivative **PSU**: Power Supply Unit **PWM**: Pulse Width Modulation **QoS**: Quality of Service **RMSE**: Root Mean Square Error **RS485**: Recommended Standard 485 **RTOS**: Real-Time Operating System **SPI**: Serial Peripheral Interface **SPIFFS**: SPI Flash File System **TSP**: Traveling Salesman Problem **UART**: Universal Asynchronous Receiver-Transmitter **USB**: Universal Serial Bus **VRA**: Variable-Rate Application **WiFi**: Wireless Fidelity **YOLO**: You Only Look Once.
